# Catecholamines Promote *Actinobacillus pleuropneumoniae* Growth by Regulating Iron Metabolism

**DOI:** 10.1371/journal.pone.0121887

**Published:** 2015-04-07

**Authors:** Lu Li, Zhaohui Chen, Weicheng Bei, Zhipeng Su, Qi Huang, Liang Zhang, Huanchun Chen, Rui Zhou

**Affiliations:** State Key Laboratory of Agricultural Microbiology, College of Veterinary Medicine, Huazhong Agricultural University, Wuhan 430070, China; East Carolina University School of Medicine, UNITED STATES

## Abstract

Catecholamines are host stress hormones that can induce the growth of many bacteria by facilitating iron utilization and/or regulate the expression of virulence genes through specific hormone receptors. Whether these two responsive pathways are interconnected is unknown. In our previous study, it was found that catecholamines can regulate the expression of a great number of genes of *Actinobacillus pleuropneumoniae*, an important swine respiratory pathogen. However, bacterial growth was not affected by catecholamines in rich medium. In this study, it was discovered that catecholamines affected *A*. *pleuropneumoniae* growth in chemically defined medium (CDM). We found that serum inhibited *A*. *pleuropneumoniae* growth in CDM, while epinephrine, norepinephrine and dopamine promoted *A*. *pleuropneumoniae* growth in the CDM containing serum. The known bacterial hormone receptor QseC didn’t play roles in this process. Ion-supplementation and transcriptome analysis indicated that serum addition resulted in iron-restricted conditions which were alleviated by the addition of catecholamines. Transferrin, one of the components in serum, inhibited the growth of *A*. *pleuropneumoniae* in CDM, an effect reversed by addition of catecholamines in a TonB2-dependent manner. Our data demonstrate that catecholamines promote *A*. *pleuropneumoniae* growth by regulating iron-acquisition and metabolism, which is independent of the adrenergic receptor QseC.

## Introduction

In recent years, studies in microbial endocrinology have discovered that host stress-related neuroendocrine catecholamine hormones can activate pathogen responses [1], providing an important bridge between infectious disease and stress. In 1992, the first study confirming the direct effect of catecholamines on bacterial growth was reported [2]. After that, many studies have discovered that catecholamines can stimulate the growth of bacteria [3–7]. Meanwhile, other studies have reported that in different bacterial species, catecholamines can regulate the expression of lots of genes including those involved in virulence [8–13]. Bacterial colonists of the gut, oral cavity and respiratory tract are exposed and can respond to catecholamines [1]. In some bacteria, catecholamines can facilitate the capture of iron from host transferrin (Tf) and lactoferrin (Lf) and promote bacterial growth via an enterobactin uptake system [14,15]. Norepinephrine (NE) can form complexes with the iron within Tf / Lf and reduce Fe^3+^ to Fe^2+^, resulting in release of the iron from the siderophores [16]. In other studies, the sensor kinases QseC and QseE, which are members of bacterial two component signal transduction systems (TCSTS), have been identified as adrenergic receptors in *Escherichia coli* O157:H7 [17–19]. QseC, QseE and their cognate regulators form complicated signaling cascades, linking host hormone responses to *E*. *coli* virulence [17–19]. However, it is still uncertain that whether there is any connection between the catecholamine- induced growth and QseC/E dependent signaling pathways.

*Actinobacillus pleuropneumoniae*, a member of the family *Pasteurellaceae*, is the etiologic agent of porcine contagious pleuropneumonia causing substantial global economic losses in the pig industry [20]. Stress factors including crowding, transportation, movement of pigs and adverse climatic conditions, contribute to *A*. *pleuropneumoniae* infection and transmission [21]. Following such stresses, the morbidity and mortality of disease are consequently affected [21]. Iron-acquisition systems are important factors involved in *A*. *pleuropneumoniae* infection [22]. *A*. *pleuropneumoniae* can use various iron sources from the host. Porcine Tf and haem compounds, but not porcine Lf, can be used by *A*. *pleuropneumoniae* as a sole iron source [23–26]. No siderophore has been found in *A*. *pleuropneumoniae*, but this bacterium can use exogenously supplied hydroxamate and catechol siderophores to promote growth [27]. *A*. *pleuropneumoniae* has two TonB systems encoded by *tonB1-exbB-exbD* and *tonB2-exbB2-exbD2* [28]. TonB2 has been confirmed to be essential for infection in the host [28]. *afuABC* encoding a ferric uptake ABC transporter [29], *hbpA* encoding a hemoglobin binding protein [30], and *fhuABCD* encoding a ferrichrome transporter [31] have also been reported in *A*. *pleuropneumoniae*. Genome sequencing has identified at least 55 genes in the *A*. *pleuropneumoniae* genome that are involved in iron acquisition and metabolism [32]. The iron-acquisition and metabolism genes of *A*. *pleuropneumoniae*, including the two *tonB* gene systems, are up-regulated when bacteria are grown under iron-restriction [28,33].

Our previous study found that *A*. *pleuropneumoniae* can actively respond to the host stress hormones epinephrine (Epi) and NE [34]. The two hormones can affect expression of a great number of genes involved in *A*. *pleuropneumoniae* infection and metabolic processes. One of the genes regulated by the hormones was *qseC*, encoding the known adrenergic receptor QseC. The effects on selected virulence determinants were also found. However, in our previous study, catecholamines had no effect on *A*. *pleuropneumoniae* growth in rich medium. To further understand the response of *A*. *pleuropneumoniae* to catecholamines, in this study, we investigated the response of the bacterium to stress hormones when grown in chemically defined medium (CDM). Investigations included the iron-utilization mechanisms involved and whether they were dependent on the QseC pathway.

## Materials and Methods

### Bacterial strains and culture conditions

*A*. *pleuropneumoniae* 4074 (serovar 1 reference strain) and its mutants *ΔqseB*, *ΔqseC*, *ΔqseBC* (constructed by deletion inactivation using a sucrose counter-selectable marker system as described previously [35]) and *ΔtonB1*, *ΔtonB2* (reported in previous study [28]) were used in this study. Tryptic Soy Broth (TSB) (Difco Laboratories, USA) or Tryptic Soy Agar (TSA) (Difco Laboratories, USA) supplemented with 10 μg/ml of nicotinamide adenine dinucleotide (NAD) and 10% (v/v) filtered cattle serum was used as rich medium. For iron-restricted conditions in rich medium, 2,2-dipyridyl at 100μM was added into the TSB medium [36]. The chemically defined medium (CDM) was prepared as previously described [37]. Filtered cattle serum, FeCl_3_, MgCl_2_, ZnCl_2_, apo-transferrin (ATF) or holo-transferrin (HTF) (Sigma, USA) at various concentrations was added into the CDM when necessary. For detection of the effects of catecholamines and/or their antagonists on bacterial growth, Epi, NE and dopamine (DA), α-adrenergic receptor antagonist phentolamine (PE), β- adrenergic receptor antagonist propranolol (PO) and the non-selective dopaminergic receptor antagonist haloperidol (Hal) (Sigma, USA) [38] were supplemented into the medium. To assess bacterial growth, bacteria were cultured aerobically at 37°C with rotation at 200 rpm and optical density at 600_nm_ (OD_600nm_) and/or bacterial viable numbers were recorded at selected time points.

### Microarray construction

The microarray used in this study has been described previously [34]. In brief, the microarray consists of 15744 60-mer oligonucleotide probes synthesized *in situ* by Agilent Technologies. The probes were designed based on the genome sequences of *A*. *pleuropneumoniae* 4074 (serovar 1), JL03 (serovar 3) and L20 (serovar 5) (GenBank accession numbers: AACK00000000, CP000687, CP000569) including 2132 ORFs. Each probe with the same sequence for a given gene was repeated twice on the array.

### Microarray experiments and data analysis

The bacterial culture samples were collected from mid-log phase cultures (7 hours after culturing with an inoculum of 10^6^ CFU/ml in CDM). Three independent biological replicates were performed. Total RNA was extracted using RNA-Solv Reagent (Promega, USA) according to the manufacturer’s instructions. Hybridization and scanning were conducted according to the Agilent microarray single channel experiment protocols (Agilent, USA).

The signal intensities were normalized using Feature Extraction Software (Agilent, USA) and transformed into log2 values. The genes with positive signals (flags = P or M) in all hybridizations were selected to be further analyzed. The genes with fold change ≥ 2 and *P* < 0.05 were selected as differentially expressed genes. Gene annotations and functional classification were conducted according to our previous studies [34,39]. All the data are MIAME compliant and the raw data has been deposited in the NCBI GEO database under the number GSE61054.

### Real-time quantitative RT-PCR

RNA was extracted as described above and reverse-transcribed into cDNA using Superscript II reverse transcriptase (Invitrogen, USA). Real time quantitative RT-PCR (qRT-PCR) was performed using ABI Power SYBR Green PCR Master Mix (ABI, USA) and the 7900 HT Sequence Detection System (ABI, USA) at 50°C, 5 min; 95°C, 10 min; 40 cycles of 95°C, 15 s; 60°C, 1 min. The primers used for real-time qRT-PCR are listed in S1 Table. The relative transcription level of each gene was determined by normalization to that of the *kdsB* gene which displayed no changes in the present microarray analysis using the 2^-ΔCtΔCt^ method [40].

### Statistical methods

The bacterial densities revealed by OD_600nm_ values or bacterial numbers at different time-points during growth were compared by using a two-tailed paired student’s *t*-test. Microarray data were analyzed using the two class paired *t*-test in SAM (significance analysis of microarray) inserted into the software TM4. A two-tailed paired student’s *t*-test was used to analyze the results of qRT-PCR. The correlation between the results of microarray and qRT-PCR was determined by calculating R^2^ using the mean log2 ratios.

## Results

### Serum inhibited *A*. *pleuropneumoniae* growth in CDM

**Fig 1:**
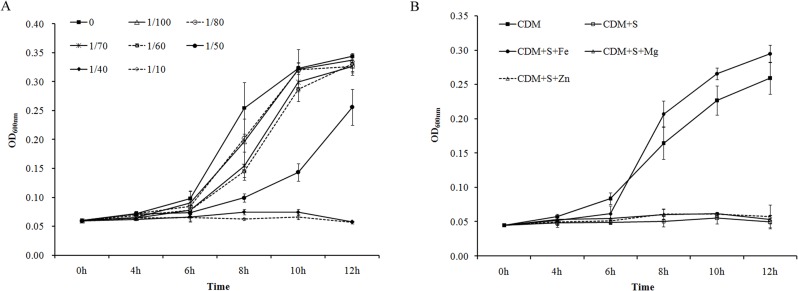
The effect of serum on *A*. *pleuropneumoniae* growth in chemically defined medium (CDM). *A*. *pleuropneumoniae* was cultured in TSB medium overnight and then sub-cultured into CDM at a dose of 10^4^ CFU/ml. Optical densities of bacterial cultures (OD_600nm_) were recorded at selected time points. **(A)** Various concentrations of serum (0–1/10; V/V) were added into CDM; **(B)** Serum at the concentration of 1/40 was added into CDM (CDM + S). FeCl_3_, MgCl_2_ and ZnCl_2_ were supplemented into the serum-containing medium respectively (CDM + S + Fe/Mg/Zn). *A*. *pleuropneumoniae* cultured in CDM without any supplementation was used as a control (CDM). Data are shown as means ± SD from four independent replications.

In our previous studies, it was found that cattle serum promoted the growth of *A*. *pleuropneumoniae* in the rich medium TSB, and catecholamines had no detectable effect on *A*. *pleuropneumoniae* growth [34,41]. To investigate the effect of catecholamines on growth under iron-limiting conditions, 2,2-dipyridyl was added to chelate the iron in the TSB medium. There was impairment of *A*. *pleuropneumoniae* growth when 2,2-dipyridyl was added into TSB in both the absence and presence of supplemented catecholamines (data not shown). In CDM,*A*. *pleuropneumoniae* grew slowly, and cattle serum was added to facilitate bacterial growth. However, unexpectedly, serum inhibited *A*. *pleuropneumoniae* growth in CDM in a concentration-dependent manner (Fig 1A). Since the concentration-dependent inhibition was similar to that resulting from addition of 2,2-dipyridyl to rich medium, iron (in the form of FeCl_3_) was added into the serum supplemented CDM to identify the possible reason of growth inhibition. Compared with MgCl_2_ and ZnCl_2_, only FeCl_3_ addition reversed serum-induced growth inhibition in CDM. Various concentrations of metal ions were tested and the similar results were observed. The influence of metal ions at a concentration of 40 μM on *A*. *pleuropneumoniae* growth are shown in Fig 1B.

### Catecholamines stimulated *A*. *pleuropneumoniae* growth in serum-supplemented CDM

**Fig 2:**
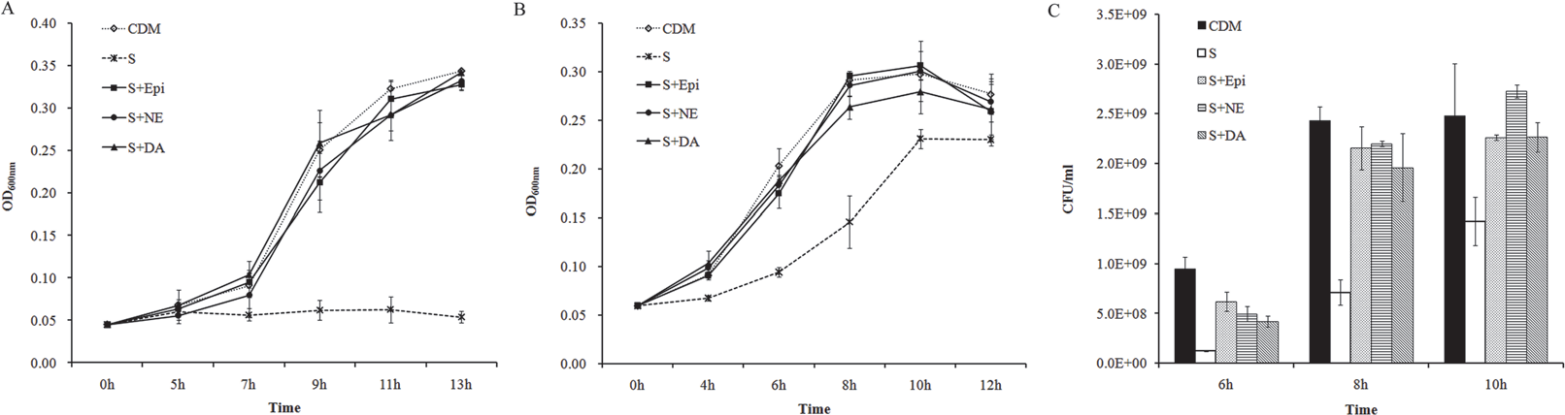
Catecholamine induced *A*. *pleuropneumoniae* growth in CDM containing serum. *A*. *pleuropneumoniae* was cultured in TSB medium overnight and then sub-cultured into CDM containing 1/40 of serum (S) using an inoculation dose of 10^4^ CFU/ml **(A)** or 10^6^ CFU/ml **(BC)**. Epi, NE and DA at 50μM were supplemented into the serum-containing medium (S + Epi/NE/DA). *A*. *pleuropneumoniae* cultured in CDM without any supplementation was used as control (CDM). Optical densities of bacterial cultures (OD_600nm_) were recorded at selected time points **(AB)**. Bacterial viable numbers were recorded after sub-culturing using 10^6^ CFU/ml at 6 h, 8h and 10h, respectively **(C)**. Data are shown as means ± SD from four independent replications.

The catecholamines Epi, NE and DA at the concentration of 50 μM were added into serum-supplemented CDM and growth curves obtained. The three hormones stimulated *A*. *pleuropneumoniae* growth significantly (Fig 2). With an initial inoculum of 10^4^ CFU/ml, *A*. *pleuropneumoniae* could hardly grow in the serum-supplemented CDM, but addition of catecholamines induced bacterial growth (Fig 2A). With a starting inoculum of 10^6^ CFU/ml, *A*. *pleuropneumoniae* grew in serum medium but reached a lower bacterial density in log-phase compared to that in medium containing catecholamines (Fig 2BC). Furthermore, various concentrations, ranging from 0.1 μM to 50 μM, of catecholamines were tested to detect the minimum effective concentration. The results showed that catecholamines at the concentrations higher than 0.5 μM could induce growth (S1 Fig).

### Stimulation of growth was not mediated by known adrenergic receptors

To explore the cause of growth promotion, the known adrenergic receptors in mammalian as well as in bacteria were investigated. The eukaryotic α-adrenergic receptor antagonist PE, β-adrenergic receptor antagonist PO and the non-selective dopaminergic receptor antagonist Hal were added into the medium to determine whether these antagonists could block the growth-inducing effect of catecholamines or not. A range of concentrations from 10–100 μM were used, but the antagonists did not block the growth induction caused by Epi, NE and DA. The results using concentration of 50μM are shown in S2 Fig. These observations demonstrate that the mechanism of growth promotion by catecholamines did not involve the eukaryotic adrenergic signaling pathways investigated.

The TCSTS sensor kinases QseC and QseE are known as adrenergic receptors in *E*. *coli*. *qseC* and its cognate regulator *qseB* are present in the *A*. *pleuropneumoniae* genome and are regulated by Epi and NE [34]. Thus, the deletion mutants *ΔqseC*, *ΔqseB* and *ΔqseBC* were used to determine whether growth induction was mediated by this two component system or not. However, the growth of the three mutants did not show any difference to that of the parental strain (S3 Fig), indicating that the induced growth by catecholamines in *A*. *pleuropneumoniae* was not mediated by the known bacterial adrenergic receptor QseC.

### Iron availability and metabolism contributed to the growth stimulation

To further investigate the mechanism of serum-inhibited and catecholamine-induced growth of *A*. *pleuropneumoniae* in CDM, the gene expression profiles of the bacteria grown in CDM, CDM supplemented with serum and CDM supplemented with serum plus Epi/NE/DA were compared. The microarray data showed that 363 genes (172 induced and 191 repressed) were differentially expressed in CDM supplemented with and without serum. These genes will be described as serum-regulated. Meanwhile, 255 genes (139 induced and 116 repressed), 723 genes (336 induced and 387 repressed) and 716 genes (339 induced and 377 repressed) were differentially expressed after addition of Epi, NE and DA into the serum-supplemented CDM, respectively. These genes will be described as hormone-regulated. Five genes (three genes regulated by serum and hormones, and two genes which were not regulated) were selected to conduct the qRT-PCR to validate the microarray results. The two methods showed high correlation with R^2^ = 0.903 (S4 Fig). The functions of all the differentially expressed genes can be divided into 21 categories (S5 Fig). Only a few genes involved in cell division and cell cycle were differentially expressed, while a large amount of regulated genes were metabolism-related. Among the serum-, Epi-, NE- and DA-regulated genes, an important proportion are involved in inorganic ion transport and metabolism, and 21 of these genes were found to be involved in iron metabolism. Twenty out of 21 of these iron metabolism genes were up-regulated by supplementation of serum, but down-regulated by addition of Epi, NE or DA into the serum-containing medium (Table 1). Genes encoding the two TonB systems involved in iron uptake systems of *A*. *pleuropneumoniae*, the TonB1-ExbB-ExbD and TonB2-ExbB2-ExbD2, were differentially expressed. In addition, genes encoding proteins involved in acquisition of various forms of iron including the Tf, hemoglobin, heme and ferrichrome were regulated.

**Table 1:** Differentially expressed genes encoding iron metabolism proteins regulated by serum, Epi, NE and DA.

Gene locus_tag	Name	Function	Serum		Epi		NE		DA	
			Fold change	p-value	Fold change	p-value	Fold change	p-value	Fold change	p-value
APJL_0076	*tonB2*	TonB energy transducing protein	13.33	1.62E-05	0.14	3.68E-02	0.07	1.76E-02	0.06	2.36E-02
APJL_0077	*exbD2*	biopolymer transport protein	11.80	2.37E-04	0.10	3.41E-03	0.10	9.62E-03	0.08	1.64E-02
APJL_0078	*exbB2*	biopolymer transport protein	12.75	7.75E-07	0.09	3.50E-03	0.08	9.77E-03	0.07	8.86E-03
APJL_0128	*yfeD*	iron (chelated) transport system membrane protein	2.16	2.51E-04	0.43	3.02E-04	0.22	3.85E-03	0.20	2.72E-04
APJL_0286	*frpB*	iron-regulated outer membrane protein	7.88	4.39E-05	0.09	5.10E-03	0.11	2.12E-02	0.10	1.27E-02
APJL_0554	*cirA*	outer membrane receptor proteins, mostly Fe transport	3.42	1.68E-03	0.32	3.94E-03	0.35	7.76E-03	0.27	4.58E-03
APJL_0665	*-*	high-affinity Fe^2+^/Pb^2+^ permease	5.71	2.00E-02	0.13	1.74E-02	0.21	1.75E-02	0.17	1.03E-02
APJL_1065	*hgbA*	hemoglobin and hemoglobin haptoglobin-binding protein 4	3.22	1.42E-03	0.27	1.33E-03	0.21	1.36E-03	0.21	1.82E-03
APJL_1066	*hugZ*	heme iron utilization protein	44.51	1.52E-04	0.01	1.88E-02	0.02	9.44E-06	0.01	1.20E-04
APJL_1312	*-*	iron-regulated outer membrane protein	11.63	4.10E-04	0.08	1.96E-02	0.15	1.84E-02	0.11	1.01E-02
APJL_1597	*tbpA1*	transferrin-binding protein 1	4.00	1.03E-02	0.29	3.98E-02	0.23	5.24E-03	0.25	8.63E-03
APJL_1598	*tbpB1*	transferrin-binding protein 2	5.12	7.87E-04	0.18	3.36E-04	0.17	2.97E-03	0.20	9.98E-04
APJL_1599	*exbD*	biopolymer transport protein	6.26	3.84E-03	0.12	2.19E-03	0.14	1.49E-04	0.11	5.66E-04
APJL_1600	*exbB*	biopolymer transport protein	6.38	1.21E-04	0.16	1.59E-04	0.14	6.34E-04	0.11	1.19E-03
APJL_1601	*tonB1*	periplasmic protein	5.81	9.66E-04	0.18	2.00E-02	0.18	1.76E-02	0.22	4.21E-07
APJL_1922	*-*	outer membrane receptor proteins, mostly Fe transport	0.17	4.12E-03	2.27	1.68E-02	3.20	7.60E-03	3.68	5.09E-06
APJL_2000	*-*	hemoglobin receptor precursor	12.63	9.85E-06	0.12	2.19E-02	0.09	7.86E-04	0.08	2.39E-05
APJL_2060	*hbpA2*	heme-binding protein A	4.53	1.21E-04	0.22	1.25E-02	0.28	2.66E-04	0.23	3.04E-02
APJL_2064	*fhuD*	ferrichrome-binding periplasmic protein	2.74	2.61E-03	0.41	3.27E-03	0.36	2.31E-03	0.32	1.68E-03
APJL_2065	*fhuB*	ferrichrome uptake protein	4.30	2.76E-03	0.21	6.56E-04	0.21	8.77E-04	0.17	3.32E-03
APJL_2066	*fhuA*	outer membrane ferric hydroxamate receptor	3.22	2.69E-04	0.38	4.97E-02	0.26	2.66E-02	0.24	6.53E-03

### Transferrin contributed to the growth stimulation by catecholamines

**Fig 3:**
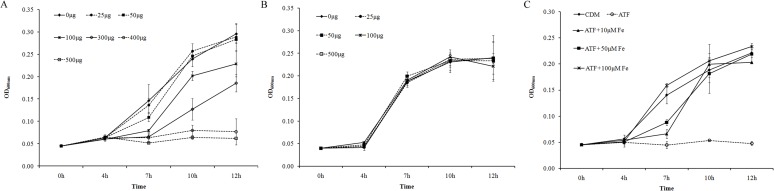
The effect of transferrin on *A*. *pleuropneumoniae* growth in CDM). *A*. *pleuropneumoniae* was cultured in TSB medium overnight and then sub-cultured into CDM at an inoculation dose of 10^4^ CFU/ml. Optical densities of bacterial cultures (OD_600nm_) were recorded at selected time points. Various concentrations of apo-transferrin (ATF) **(A)** or holo-transferrin (HTF) **(B)** were added into CDM. **(C)**. ATF at 400μg/ml was added into CDM (ATF) and FeCl_3_ at different concentrations were supplemented into the medium containing ATF (ATF + Fe). *A*. *pleuropneumoniae* cultured in CDM without any supplementation was used as a control (CDM). Data are shown as means ± SD from four independent replications.

Tf has been identified as the key component in serum that stimulates the growth of *E*. *coli* [42]. *A*. *pleuropneumoniae* can also use Tf as an iron source [28]. Thus, both ATF and HTF were added into CDM (instead of the serum) to further identify the cause of growth promotion by catecholamines in *A*. *pleuropneumoniae*. ATF inhibited *A*. *pleuropneumoniae* growth in a concentration-dependent manner while the HTF had no effect (Fig 3AB). ATF contains no iron but HTF is saturated with iron. Hence, we added both ATF and FeCl_3_ into the CDM. The growth curves showed that with the increased amount of FeCl_3_, the inhibition effect of ATF on *A*. *pleuropneumoniae* growth disappeared (Fig 3C). Hence, the inhibition of ATF on *A*. *pleuropneumoniae* growth was due to iron availability.

**Fig 4:**
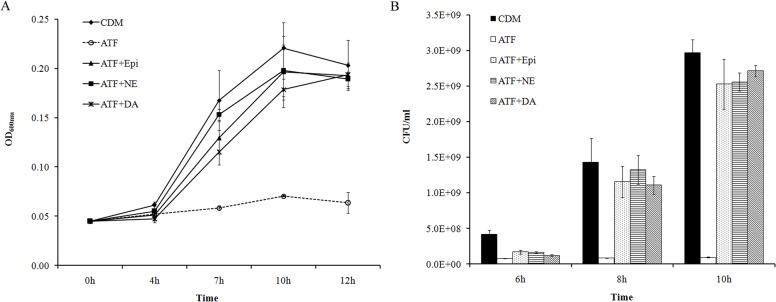
Catecholamine induced *A*. *pleuropneumoniae* growth in CDM containing ATF. *A*. *pleuropneumoniae* was cultured in TSB medium overnight and then sub-cultured into CDM using an inoculation dose of 10^4^ CFU/ml. ATF at 400μg/ml was added into CDM (ATF). Epi, NE and DA at 50μM were supplemented into the medium containing ATF (ATF + Epi/NE/DA). *A*. *pleuropneumoniae* cultured in CDM without any supplementation was used as a control (CDM). **(A)**. Optical densities of bacterial cultures (OD_600nm_) were recorded at selected time points. **(B)**. Bacterial viable numbers were recorded at 6 h, 8h and 10h. Data are shown as means ± SD from four independent replications.

When catecholamines were added into CDM containing 400μg/ml ATF, the growth of *A*. *pleuropneumoniae* was totally inhibited. When the hormones Epi, NE or DA were added into CDM containing 400μg/ml ATF, the growth of *A*. *pleuropneumoniae* was promoted (Fig 4). In contrast, catecholamines did not show any effect on bacterial growth in the CDM containing HTF (data not shown). Furthermore, under such growth conditions, the eukaryotic adrenergic receptor antagonists did not block the growth stimulation, and the *A*. *pleuropneumoniae ΔqseC*, *ΔqseB* and *ΔqseBC* mutants showed the same growth characteristics as their parental strain (data not shown). Thus, it can be concluded that Tf is one of the components in serum that contributes to catecholamine-induced growth of *A*. *pleuropneumoniae*. Again, this growth stimulation is not mediated by the known adrenergic receptors investigated.

### TonB2 contributed to growth stimulation of *A*. *pleuropneumoniae* by catecholamines

**Fig 5:**
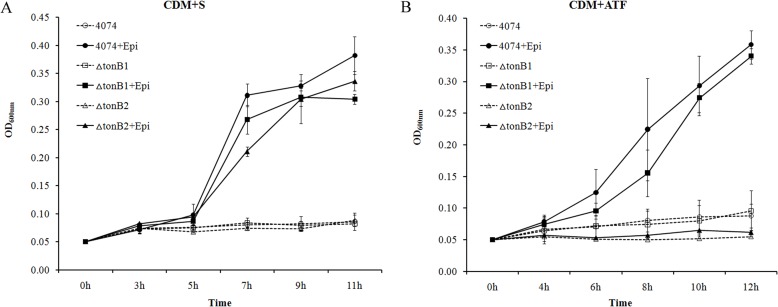
The effects of catecholamines on growth of *A*. *pleuropneumoniae ΔtonB1* and *ΔtonB2* mutants. *A*. *pleuropneumoniae* was cultured in TSB medium overnight and then sub-cultured into CDM using an inoculation dose of 10^4^ CFU/ml. *A*. *pleuropneumoniae* 4074 and the mutants were sub-cultured in CDM containing 50μM of Epi and 1/40 of serum (S in figure A) or 400μg/ml ATF (figure B). Optical densities of bacterial cultures (OD_600nm_) were recorded at selected time points. Data are shown as means ± SD from four independent replications.

According to the results of microarray analysis, multiple genes involved in iron-acquisition, including *tonB1* and *tonB2*, were regulated by serum and catecholamines. *ΔtonB1* and *ΔtonB2* mutants were used to discover if either or both TonB systems have a role in growth induction by catecholamines. In serum-containing CDM, the growth of both *ΔtonB1* and *ΔtonB2* mutants were inhibited as the parental strain. The growths of the two mutants were also promoted by addition of Epi in serum-containing CDM (Fig 5A). When ATF was used to replace serum, the *ΔtonB1* mutant had similar growth features as that of the parental strain, whereas the growth of the *ΔtonB2* mutant was not induced by Epi (Fig 5B). NE and DA displayed the same effect as Epi with both mutants (data not shown). Therefore, the TonB2 system has an important role in catecholamine-induced growth in medium containing ATF.

## Discussion

In our previous study, we found that *A*. *pleuropneumoniae* alters virulence gene expression and infection-related behaviour in response to the host stress hormones Epi and NE [34]. In that work, we used the rich medium TSB to support the growth of *A*. *pleuropneumoniae*. Catecholamines had no effect on growth in the TSB. According to previous studies, bacterial growth can be induced by catecholamines as a result of iron-acquisition [14]. Therefore, in the present study, we investigated the effect of catecholamines on *A*. *pleuropneumoniae* growth under iron-restricted conditions. The addition of the Fe-chelator 2,2-dipyridyl was used to induce iron-restriction in rich medium but addition of catecholamine had no effect on *A*. *pleuropneumoniae* growth (data not shown). The use of CDM in this study allowed the effects of catecholamines on *A*. *pleuropneumoniae* to be determined. Initially, it was unexpected that, in contrast to that observed with the rich medium TSB, addition of serum to CDM resulted in growth inhibition of *A*. *pleuropneumoniae*. Analysis of the growth characteristics of *A*. *pleuropneumoniae* in media supplemented with ions in combination with gene expression changes in response to supplementation of serum and serum plus catecholamines to CDM indicated that addition of serum resulted in iron restricted conditions in CDM for *A*. *pleuropneumoniae*. In CDM plus serum, bacterial growth was inhibited and iron-acquisition genes were up-regulated. The addition of catecholamines removed the iron-restriction, hence bacterial growth was induced and iron-acquisition genes were down-regulated. Similar discoveries have also been reported in previous studies with *Salmonella enterica*, in which 36 genes involved in iron-utilization were down-regulated after the exposure to NE, confirming the iron-restricted environment resulted from the serum-SAPI medium used in that study [15].

In *E*. *coli* O157, regulation of virulence genes by catecholamines are dependent on the QseC/B and QseE/F two component systems [18]. There are no homologous genes to *qseE/F* in *A*. *pleuropneumoniae*. Homologous *qseC/B* genes are found in *A*. *pleuropneumoniae* which are regulated by Epi and NE [34]. However, mutations of *qseC/B* did not change catecholamine-induced growth of *A*. *pleuropneumoniae*, and the expression of *qseC/B* were neither regulated by serum nor catecholamines in CDM. Therefore, under the iron-restricted conditions tested in this study, catecholamines-stimulated growth of *A*. *pleuropneumoniae* was independent of QseC/B. Most previous studies investigating growth promotion effects of catecholamines have been carried out in medium containing serum or Tf [4–6,15], but those characterizing QseC/B and QseE/F signaling cascades have used different media [18,19]. The systems used by bacteria to respond to host catecholamines may vary dependent on growth conditions.

In this study, the ATF-supplemented CDM may capture iron in the medium to establish iron-restricted condition for *A*. *pleuropneumoniae*. As shown in previous study, catecholamines can form a complex with transferrin-bound iron and release the iron for the bacterium to utilize [16]. Hence the growth of *A*. *pleuropneumoniae* could be promoted by catecholamines. *A*. *pleuropneumoniae* has two TonB systems in which TonB2 is essential for survival *in vivo* [28]. In this study, we found TonB2 played an important role in catecholamine-induced growth in ATF-supplemented CDM. This TonB2 dependent adjustment of growth might be important for *A*. *pleuropneumoniae* infection in the host during stress. In *Bordetella bronchiseptica*, another respiratory pathogen, catecholamine-induced growth is also TonB-dependent [43]. In *B*. *bronchiseptica*, NE can release iron from transferrin and deliver it directly to bacterial cells or shuttle iron to siderophores. The newly characterized NE receptors and a specific siderophore receptor of *B*. *bronchiseptica* are required, respectively [43]. Similar mechanisms present in *B*. *bronchiseptica* enabling catecholamine-induced growth may also exist in *A*. *pleuropneumoniae*. It has been reported that *A*. *pleuropneumoniae* produce uncharacterized siderphores and can use exogenously supplemented hydroxamate and catechol siderophores [27]. TonB2 is crucial for the acquisition of iron in the form of hemin, hemoglobin, ferrichrome/hydroxamate and transferrin [28]. The genes encoding proteins for binding and uptake of these forms of irons have been found in the *A*. *pleuropneumoniae* genome [32]. In a recent study, genes encoding a putative enterobactin receptor system and a *cirA* like siderophore have been identified to be up-regulated under iron-restricted conditions [33,44]. These known and/or putative iron-acquisition systems might have roles in catecholamine-induced growth of *A*. *pleuropneumoniae*.

The results suggest that, in the CDM containing serum, the induced growth of *A*. *pleuropneumoniae* is TonB2 independent. There are three possible reasons: (1) Tf was not the sole component in serum that catecholamines bind and induce bacterial growth; (2) in the medium containing serum, TonB1 has the same function as TonB2; and (3) unknown iron-uptake system(s) independent of TonB have roles in iron-acquisition in medium containing serum (but not ATF). In fact, *A*. *pleuropneumoniae* is equipped with lots of genes encoding various iron-acquisition systems [32]. Many of these genes were differentially expressed after supplementation of serum and Epi/NE/DA (Table 1). Perhaps growth in serum-containing medium involves more than one iron uptake system.

## Conclusion

In conclusion, the effect of the addition of catecholamines on *A*. *pleuropneumoniae* growth was tested in CDM. In CDM, serum and ATF inhibited *A*. *pleuropneumoniae* growth in a concentration-dependent manner. This growth inhibition was reversed by the addition of the catecholamines Epi, NE and DA in a bacterial adrenergic receptor QseC-independent manner. The underlying mechanism found was that the addition of serum or ATF to CDM resulted in iron-restricted conditions and this was reversed by addition of catecholamines. TonB2 of *A*. *pleuropneumoniae* was essential for catecholamine-induced growth of *A*. *pleuropneumoniae* in the CDM containing ATF. Thus, the results show that catecholamines induce growth of *A*. *pleuropneumoniae* through iron-acquisition in a QseC-independent manner. This response may contribute to disease development caused by *A*. *pleuropneumoniae*.

## Supporting Information

S1 Fig The effects of various concentrations of catecholamines on *A*. *pleuropneumoniae* growth in CDM containing serum. *A*. *pleuropneumoniae* was cultured in TSB medium overnight and then sub-cultured into CDM using an inoculation dose of 10^4^ CFU/ml. Catecholamines at concentrations ranging from 0.1μM to 50μM were added into CDM containing 1/40 of serum. Optical densities of bacterial cultures (OD_600nm_) were recorded at early stationary phase (12 hours after sub-culture). Data are from one test out of three similar results.
(TIF)

S2 Fig The effects of eukaryotic adrenergic receptor antagonists on catecholamine-induced growth. *A*. *pleuropneumoniae* was cultured in TSB medium overnight and then sub-cultured into CDM containing 1/40 of serum (S) using an inoculation dose of 10^4^ CFU/ml. Epi **(A)**, NE **(B)** and DA **(C)** at 50μM were supplemented into the serum-containing medium. The eukaryotic α-adrenergic receptor antagonist phentolamine (PE), β- adrenergic receptor antagonist propranolol (PO) and the non-selective dopaminergic receptor antagonist haloperidol (Hal) at the concentration of 50μM were separately added into the serum medium containing different catecholamines. *A*. *pleuropneumoniae* cultured in CDM without any supplementation was used as a control (CDM). Optical densities of bacterial cultures (OD_600nm_) were recorded at selected time points. Data are shown as means ± SD from three independent replications.
(TIF)

S3 Fig The effects of catecholamines on growth of *A*. *pleuropneumoniae ΔqseC* and *ΔqseB* mutants in CDM containing serum. *A*. *pleuropneumoniae* parental strain 4074 and the mutants (*ΔqseB* and *ΔqseC*) were cultured in TSB medium overnight and then sub-cultured in CDM containing 1/40 of serum with or without 50μM of different catecholamines (+ Epi/NE/DA). The inoculation dose was 10^4^ CFU/ml for sub-culture. Optical densities of bacterial cultures (OD_600nm_) were recorded at selected time points. Data are shown as means ± SD from three independent replications.
(TIF)

S4 Fig Validation of microarray analysis by real time qRT-PCR. Mean log2 ratios obtained from microarray results are plotted against the mean log2 ratios obtained from qRT-PCR. 1–4: gene *tonB2* regulated by serum, Epi, NE and DA; 5–8: gene *tonB1* regulated by serum, Epi, NE and DA, 9–12: gene *tbpA1* regulated by serum, Epi, NE and DA; 13–16: gene *qseC* regulated by serum, Epi, NE and DA; 17–20: gene *fur* regulated by serum, Epi, NE and DA.
(TIF)

S5 Fig Function classification of differentially expressed genes. Up or down means up or down-regulated by serum/Epi/NE/DA. Gene functions were sorted according to COG categories: C: Energy production and conversion; D: Cell cycle control, cell division, chromosome partitioning; E: Amino acid transport and metabolism; F: Nucleotide transport and metabolism; G: Carbohydrate transport and metabolism; H: Coenzyme transport and metabolism; I: Lipid transport and metabolism; J: Translation, ribosomal structure and biogenesis; K: Transcription; A, RNA processing and modification; L: Replication, recombination and repair; M: Cell wall/membrane/envelope biogenesis; O: Posttranslational modification, protein turnover, chaperones; P: Inorganic ion transport and metabolism; Q: Secondary metabolites biosynthesis, transport and catabolism; R: General function prediction only; T: Signal transduction mechanisms; U: Intracellular trafficking, secretion, vesicular transport; V: Defense mechanisms; S/N: Function unknown in COG.
(TIF)

S1 Table Primers used in this study. (DOCX)
