# Supplementary figures and images for: Catecholamines Promote Actinobacillus pleuropneumoniae Growth by Regulating Iron Metabolism
Source: PLoS One. 2015 Apr 7;10(4):e0121887. doi: 10.1371/journal.pone.0121887 (PMC4388731; doi:10.1371/journal.pone.0121887)

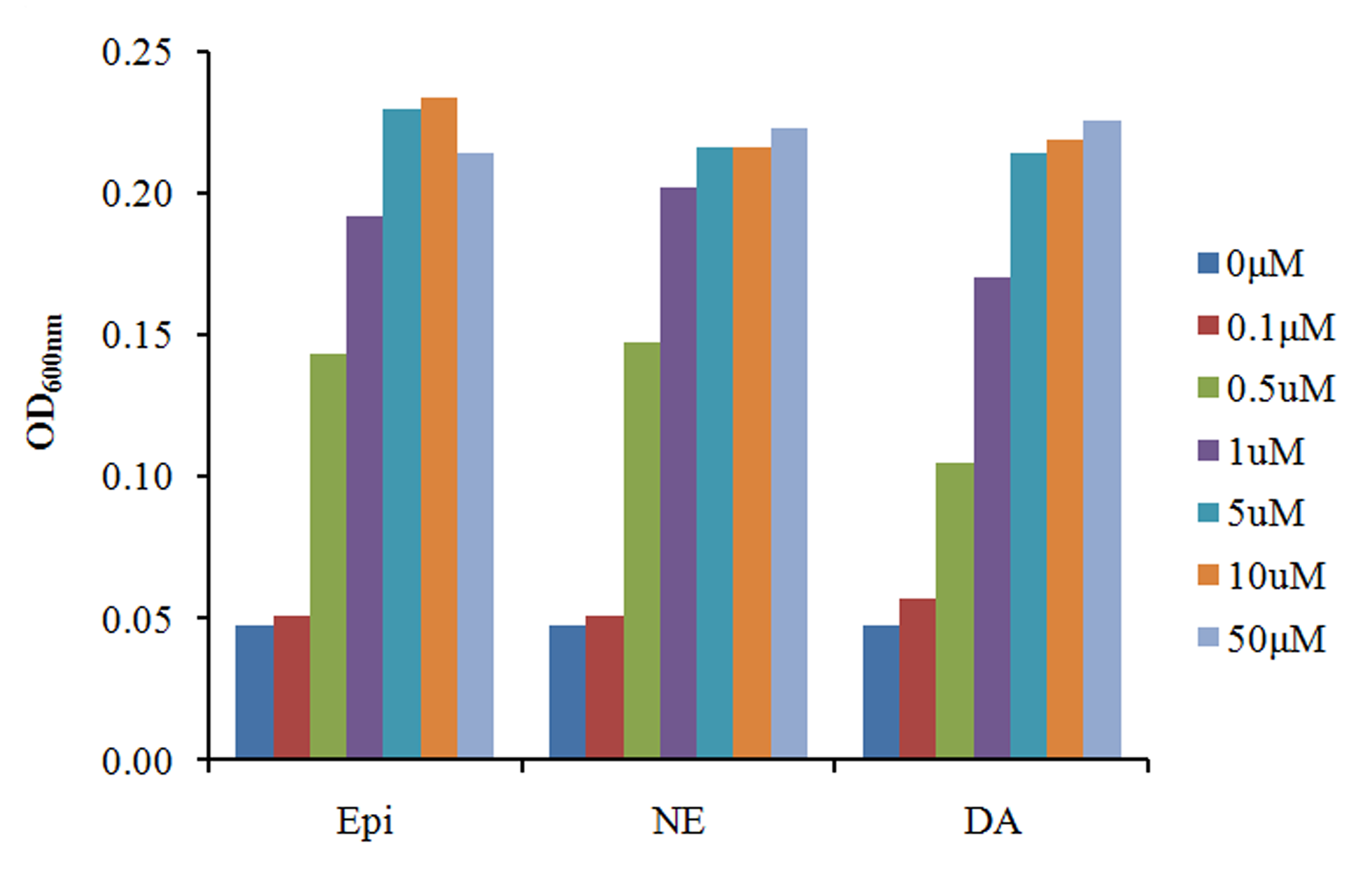

Supplement: S1 Fig — A. pleuropneumoniae was cultured in TSB medium overnight and then sub-cultured into CDM using an inoculation dose of 104 CFU/ml. Catecholamines at concentrations ranging from 0.1μM to 50μM were added into CDM containing 1/40 of serum. Optical densities of bacterial cultures (OD600nm) were recorded at early stationary phase (12 hours after sub-culture). Data are from one test out of three similar results. (TIF) [file pone.0121887.s001.tif]

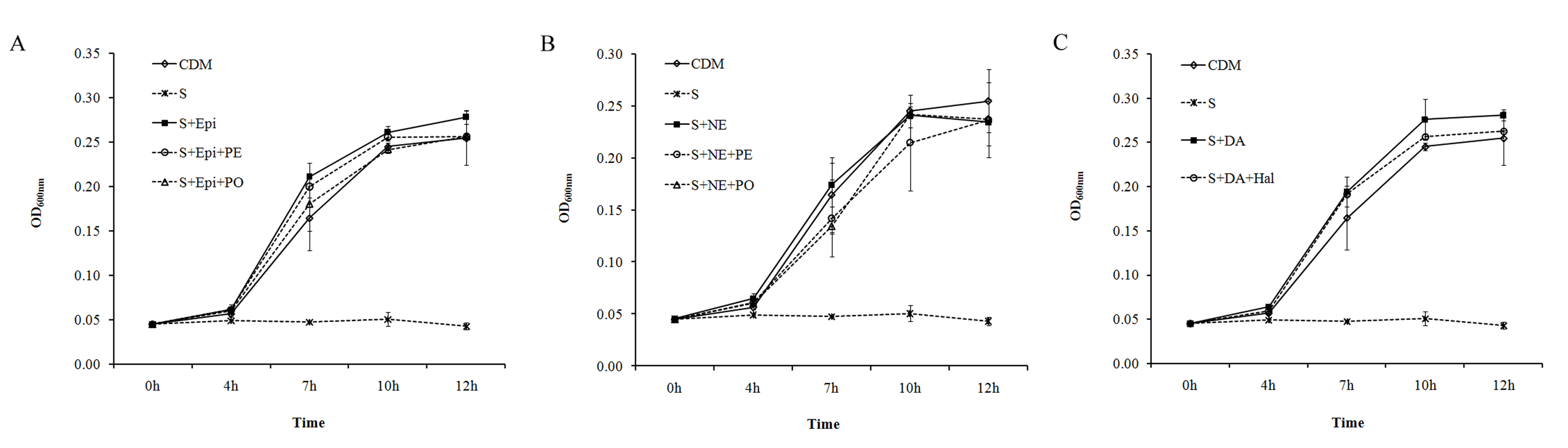

Supplement: S2 Fig — A. pleuropneumoniae was cultured in TSB medium overnight and then sub-cultured into CDM containing 1/40 of serum (S) using an inoculation dose of 104 CFU/ml. Epi (A), NE (B) and DA (C) at 50μM were supplemented into the serum-containing medium. The eukaryotic α-adrenergic receptor antagonist phentolamine (PE), β- adrenergic receptor antagonist propranolol (PO) and the non-selective dopaminergic receptor antagonist haloperidol (Hal) at the concentration of 50μM were separately added into the serum medium containing different catecholamines. A. pleuropneumoniae cultured in CDM without any supplementation was used as a control (CDM). Optical densities of bacterial cultures (OD600nm) were recorded at selected time points. Data are shown as means ± SD from three independent replications. (TIF) [file pone.0121887.s002.tif]

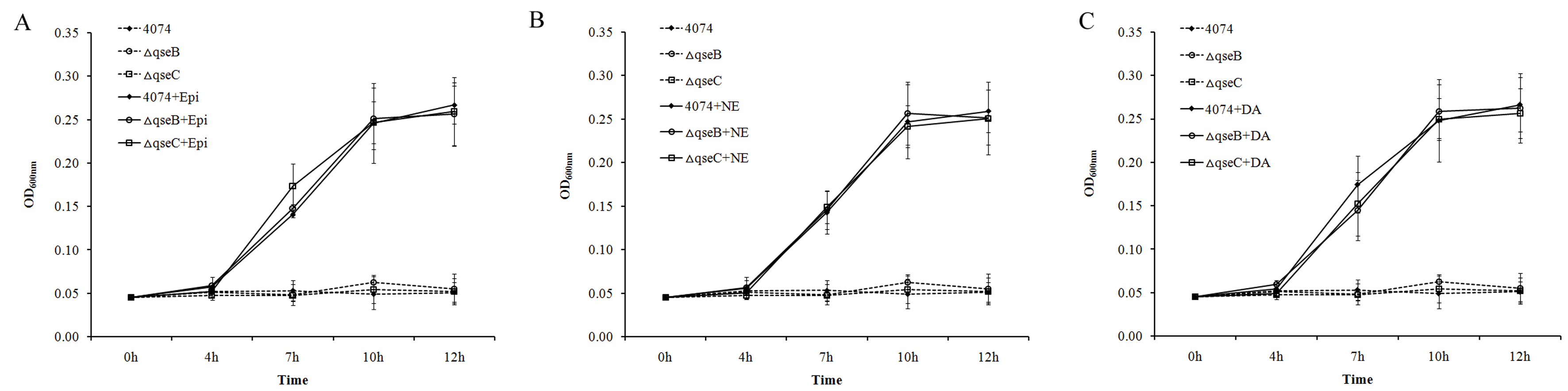

Supplement: S3 Fig — A. pleuropneumoniae parental strain 4074 and the mutants (ΔqseB and ΔqseC) were cultured in TSB medium overnight and then sub-cultured in CDM containing 1/40 of serum with or without 50μM of different catecholamines (+ Epi/NE/DA). The inoculation dose was 104 CFU/ml for sub-culture. Optical densities of bacterial cultures (OD600nm) were recorded at selected time points. Data are shown as means ± SD from three independent replications. (TIF) [file pone.0121887.s003.tif]

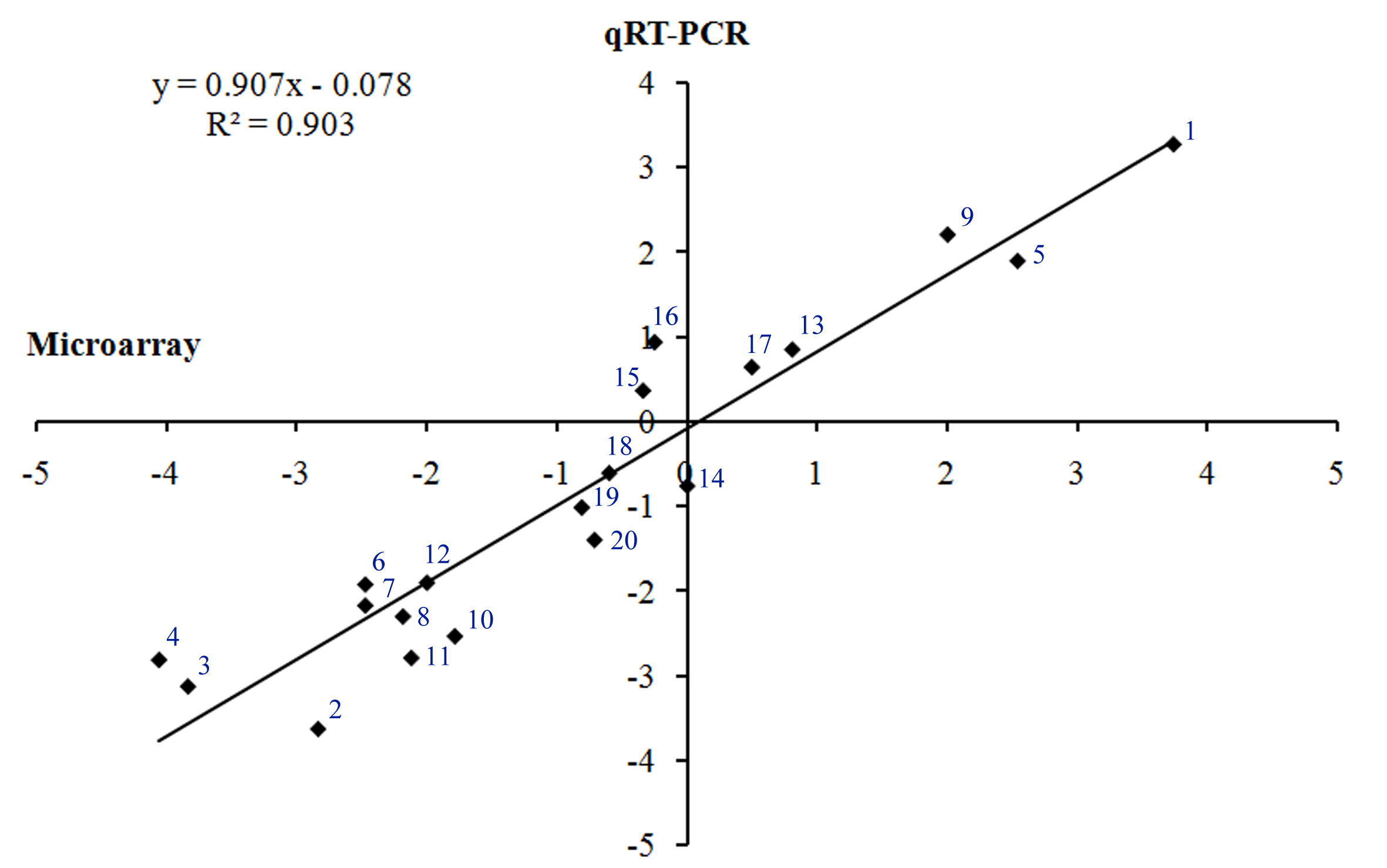

Supplement: S4 Fig — Mean log2 ratios obtained from microarray results are plotted against the mean log2 ratios obtained from qRT-PCR. 1–4: gene tonB2 regulated by serum, Epi, NE and DA; 5–8: gene tonB1 regulated by serum, Epi, NE and DA, 9–12: gene tbpA1 regulated by serum, Epi, NE and DA; 13–16: gene qseC regulated by serum, Epi, NE and DA; 17–20: gene fur regulated by serum, Epi, NE and DA. (TIF) [file pone.0121887.s004.tif]

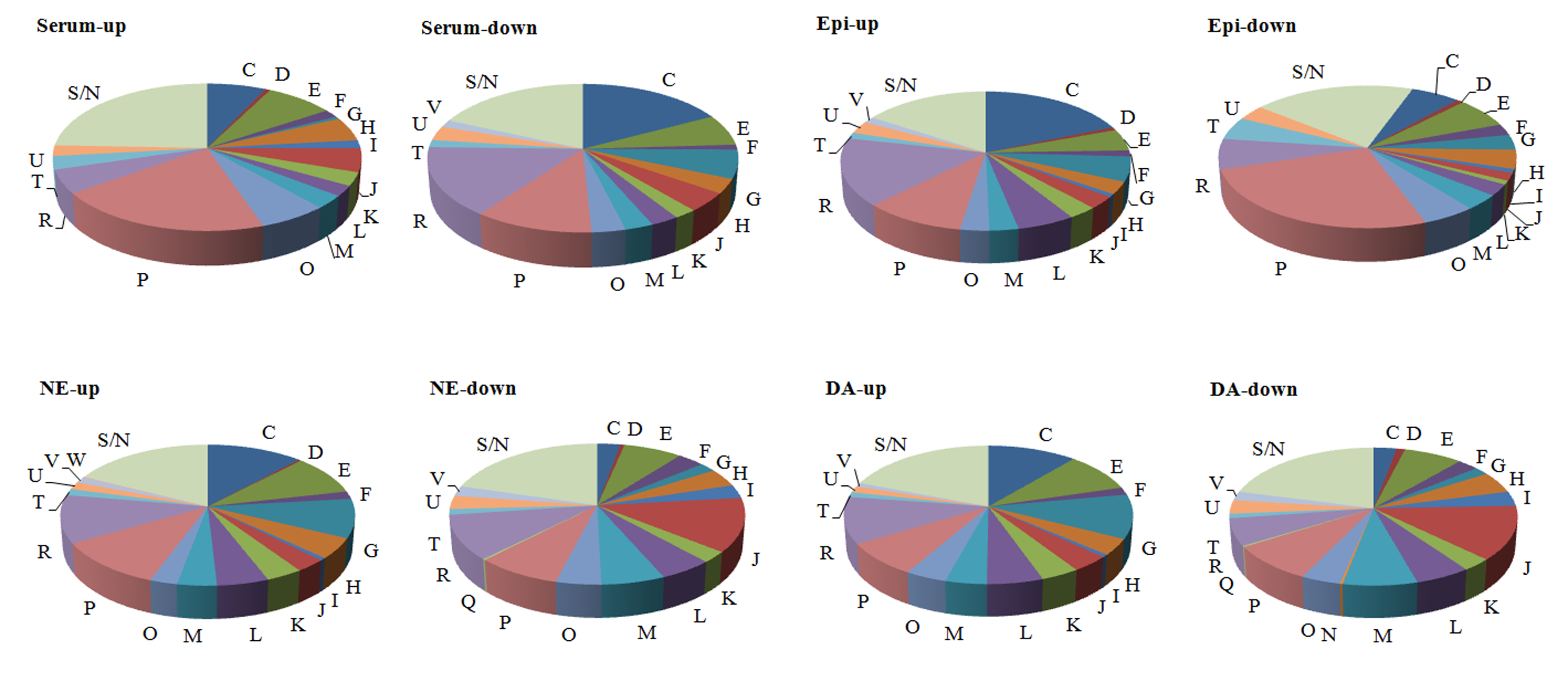

Supplement: S5 Fig — Up or down means up or down-regulated by serum/Epi/NE/DA. Gene functions were sorted according to COG categories: C: Energy production and conversion; D: Cell cycle control, cell division, chromosome partitioning; E: Amino acid transport and metabolism; F: Nucleotide transport and metabolism; G: Carbohydrate transport and metabolism; H: Coenzyme transport and metabolism; I: Lipid transport and metabolism; J: Translation, ribosomal structure and biogenesis; K: Transcription; A, RNA processing and modification; L: Replication, recombination and repair; M: Cell wall/membrane/envelope biogenesis; O: Posttranslational modification, protein turnover, chaperones; P: Inorganic ion transport and metabolism; Q: Secondary metabolites biosynthesis, transport and catabolism; R: General function prediction only; T: Signal transduction mechanisms; U: Intracellular trafficking, secretion, vesicular transport; V: Defense mechanisms; S/N: Function unknown in COG. (TIF) [file pone.0121887.s005.tif]
