# Supplementary material for: Catecholamines Promote Actinobacillus pleuropneumoniae Growth by Regulating Iron Metabolism
Source: PLoS One. 2015 Apr 7;10(4):e0121887. doi: 10.1371/journal.pone.0121887 (PMC4388731; doi:10.1371/journal.pone.0121887)
Supplement: S1 Table — (DOCX) [file pone.0121887.s006.docx]

**S1 Table. Primers used in this study**

| **Primer** | **Sequence** |
| --- | --- |
| *tonB2* | CCGCCAACGGCTCACA  ACGCACCGATTTCACTTCC |
| *tonB1* | CCCTTGGTGCTGGTTATGG  TTGATTGCTTTGCCGTTTG |
| *tbpA1* | CGTAACCGTGTTGGCTTGG  CGAACGCAGATTTTCGTATTCTAT |
| *fur* | TCTGTATGGATTGCGGTAAGGT  CCGTGCTGTTCGCTGATTT |
| *qseC* | TACCGCCACAGCGATAGAAT  CGGCAAACGCCAAAGC |
| *kdsB* | CAATCCGAATGCCGTCAAA  CGGCGCACGAGAGAAATAG |
